# Isolation and characterization of microsatellite markers from the olive fly, *Bactrocera oleae*, and their cross-species amplification in the Tephritidae family

**DOI:** 10.1186/1471-2164-9-618

**Published:** 2008-12-19

**Authors:** Antonios A Augustinos, Elias E Stratikopoulos, Eleni Drosopoulou, Evdoxia G Kakani, Penelope Mavragani-Tsipidou, Antigone Zacharopoulou, Kostas D Mathiopoulos

**Affiliations:** 1 Department of Biology, University of Patras, Patras, Greece; 2 Department of Biochemistry and Biotechnology, University of Thessaly, Thessaly, Greece; 3 Department of Biology, Aristotle University of Thessaloniki, Thessaloniki, Greece; 4 Department of Genetics and Development, Columbia University, New York, NY 10032, USA

## Abstract

**Background:**

The Tephritidae family of insects includes the most important agricultural pests of fruits and vegetables, belonging mainly to four genera (*Bactrocera, Ceratitis, Anastrepha *and *Rhagoletis*). The olive fruit fly, *Bactrocera oleae*, is the major pest of the olive fruit. Currently, its control is based on chemical insecticides. Environmentally friendlier methods have been attempted in the past (Sterile Insect Technique), albeit with limited success. This was mainly attributed to the lack of knowledge on the insect's behaviour, ecology and genetic structure of natural populations. The development of molecular markers could facilitate the access in the genome and contribute to the solution of the aforementioned problems. We chose to focus on microsatellite markers due to their abundance in the genome, high degree of polymorphism and easiness of isolation.

**Results:**

Fifty-eight microsatellite-containing clones were isolated from the olive fly, *Bactrocera oleae*, bearing a total of sixty-two discrete microsatellite motifs. Forty-two primer pairs were designed on the unique sequences flanking the microsatellite motif and thirty-one of them amplified a PCR product of the expected size. The level of polymorphism was evaluated against wild and laboratory flies and the majority of the markers (93.5%) proved highly polymorphic. Thirteen of them presented a unique position on the olive fly polytene chromosomes by *in situ *hybridization, which can serve as anchors to correlate future genetic and cytological maps of the species, as well as entry points to the genome. Cross-species amplification of these markers to eleven Tephritidae species and sequencing of thirty-one of the amplified products revealed a varying degree of conservation that declines outside the *Bactrocera *genus.

**Conclusion:**

Microsatellite markers are very powerful tools for genetic and population analyses, particularly in species deprived of any other means of genetic analysis. The presented set of microsatellite markers possesses all features that would render them useful in such analyses. This could also prove helpful for species where SIT is a desired outcome, since the development of effective SIT can be aided by detailed knowledge at the genetic and molecular level. Furthermore, their presented efficacy in several other species of the Tephritidae family not only makes them useful for their analysis but also provides tools for phylogenetic comparisons among them.

## Background

The Tephritidae family of insects includes the most important agricultural pests of fruits and vegetables. Most of them belong to four genera: *Bactrocera*, *Ceratitis*, *Anastrepha *and *Rhagoletis*. *Ceratitis *includes 89 different species. Among them, the Medfly, *Ceratitis capitata*, is the best so far studied member of the family and attacks over 350 different fruits and vegetables in tropical and sub-tropical regions [1], causing damages of hundreds of billions $ per year. *Anastrepha *is the most economically important genus of pests in the American tropics and subtropics and includes more than fifteen economically important pests [2]. *Rhagoletis *includes more than 60 described species distributed in Eurasia and the New World, several of which are important pests [3]. *Bactrocera *is among the largest genera in Tephritidae including about 500 species [4,5]. Many of them are serious pests of fruits and vegetables in different parts of the world [2]. The only member of this genus present in Europe is the olive fruit fly, *Bactrocera oleae*, the major pest of the olive fruit, with estimated damages of 5–30% of the global olive production, resulting in economic losses of about 800 million $ per year [6,7]. Quarantine orders against non-indigenous Tephritidae exist in all countries, demonstrating the appreciation of these species' destructive abilities and invasiveness success [8-12].

Currently, control of these insects is based on chemical insecticides. The Sterile Insect Technique (SIT) is the most promising, environmentally friendly method, based on the mass production and release of sterile insects into field populations. When the released males mate with the field females no progeny are produced and the field population may finally be suppressed. The appreciation of the negative effect of the released females [13] lead to the development of genetic sexing strains (GSS) [14]. Successful development of such approaches, however, presupposes an understanding of the species at the genetic, molecular and population level. Additionally, new molecular and genetic tools, such as genetic transformation, could prove very helpful since they can improve mass rearing of effective male insects. Such knowledge developed in the Medfly lead to successful SIT protocols (for a review, see [15]), whereas respective lack in the olive fly lead to fruitless attempts. In the early '70s, efforts to employ the SIT against the olive fly were unsuccessful [16], principally due to the low competitiveness of the sterile mass-reared males compared to the wild ones [17]. Several molecular and genetic studies have changed *B. oleae*'s research landscape in recent years. Among them we mention studies on population genetics [18-20], cytogenetics (for a review see [21]), sex-determining cascades [22,23] and, most notably, the successful genetic transformation [24], an achievement that gives new perspectives towards the efficient use of the SIT.

Microsatellites constitute very powerful genetic and molecular markers [25-27]. In the Medfly they have been used to identify sources of origin, invasion phenomena, to design control strategies [28-31], as well as in the genetic mapping of the species [32]. This last possibility renders microsatellite markers particularly useful in the olive fly, since several years of efforts have provided no morphological markers and therefore the development of classical genetics has been entirely hindered (Mavragani, unpublished; Zacharopoulou, unpublished). In addition, such markers can also be helpful in SIT development. For example, they have been successfully used in the analysis of mating systems in *B. dorsalis *[33] and *C. capitata *[34,35] and they can be used to detect the degree of differentiation between laboratory and wild flies, the main reason of SIT failure in the olive fly.

The present study enriches a previously described set of 15 microsatellite markers [36,19] with 16 new ones. Most of these markers were proven polymorphic, some of them were localized in the polytene chromosomes of the species and many of them were successfully cross-amplified in other Tephritidae species. Their utility in genetic studies and evolutionary comparisons is considered.

## Results and discussion

### Isolation and characterization of microsatellites from small-insert genomic libraries and enriched libraries

Thirty-four microsatellite containing clones were isolated from small-insert genomic libraries and 24 from enriched libraries, yielding a total of 36 and 26 discrete microsatellite motifs, respectively, since a few of them contained more than one microsatellite motif (Table 1). Despite the use of an equal mix of (GT)_15 _and (CT)_15 _as probes, there was a clear predominance of GT over CT repeats obtained from the small-insert library. This most likely reflects a difference in the abundance of these sequences in the genome, as has been the case in other Diptera, such as *D. melanogaster *[37-39], *D. simulans *[40], *A. gambiae *[41] and *C. capitata *(Stratikopoulos et al., submitted) [28]. In hymenoptera, CT repeats seem to be more abundant than GT repeats, as studies in *Apis mellifera *and *Bombus terrestris *reveal [42].

**Table 1:** Microsatellite loci motif and *in situ *localization to polytene chromosomes

	**Accession number**	**Locus name**	**Motif**	**Map position**	**T (°C)**
1	EU489746	*Boms1*	(GT)_13_	-	
2	AF467831	*Boms2*	(CA)_8_TA(CA)_3_	4 (IL)	58
3	EU489747	*Boms3*	(GT)_14_ (CAA)_3_CAG(CAA)_3_	no specific signal	
4	EU489748	*Boms4*	(GT)_15_GC(GT)_3_GC(GT)_13_GA(GT)_4_	-	
5	EU489749	*Boms5*	(CA)_10_TA(CA)_2_	95 (VR)	
6	EU489750	*Boms6*	(CA)_9_ (GT)_3_GG(GT)_11_TT(GT)_2_	-	
7	EU489751	*Boms7*	(CA)_7_CC(CA)_3_	-	
8	EU489752	*Boms8*	(CA)_7_CG(CA)_5_	-	
9	EU489753	*Boms9*	(GT)_10_CTGA(GT)_3_	-	
10	AF467830	*Boms10*	(CA)_10_GA(CA)_2_	no specific signal	55–62
11	AF467832	*Boms11 (Bo-D37)*	(CA)_7_CG(CA)_3_	-	
12	EU489754	*Boms12*	(CA)_10_AACA	26 (IIL)	58
13	EU489755	*Boms13*	GTGA(GT)_10_	-	
14	EU489756	*Boms14*	(AT)_2_GT(AT)_11_	-	
15	EU489757	*Boms15*	(GT)_11_GA(GT)_2_	no specific signal	55–62
16	AF467829	*Boms16 (Bo-D46)*	(CA)_10_	42(IIR)	58
17	EU489758	*Boms17*	(TG)_13_	44(IIR)	58
18	AF467828	*Boms18*	(CA)_13_	-	
19	EU489759	*Boms19*	CAAA(CA)_10_	-	
20	EU489760	*Boms20*	(GT)_13+_	8(IL) 2 signals	58
21	AF467827	*Boms21*	GTGG(GT)_13_ATGT	73(IVL)	58
22	DQ078248	*Boms22*	CAT(CA)_7_(TA)_3_TT(TA)_5_	5(IL)	58
23	EU489761	*Boms23*	(CA)_12_	3(IL)	58
24	EU489762	*Boms24*	(GT)_12_	64(IIIR)	58
25	AF467826	*Boms25*	(GT)_12_	30(IIL), 97&100(VR)	58/62
26	EU489763	*Boms26*	(GT)_8_AT(GT)_2_	26(IIL)	58
27	AF467825	*Boms27 (Bo-D52)*	(GA)_14_	83(IVR)	58
28	EU489764	*Boms28*	(CA)_12_CT(CA)_5 _(GA)_12_GG(GA)_11_	-	
29	AF467824	*Boms29*	(GT)_10_	-	
30	AF467823	*Boms30*	(GT)_17_	5–6(IL)	58
31	DQ078249	*Boms31*	(GT)_4_GC(GT)_6_GC(GT)_2_	63&65(IIIR)	58,62
32	EU489765	*Boms32*	(CA)_14_	-	
33	EU489766	*Boms33*	(CA)_2_TA(CA)_9_	no signal	55–58
34	EU489767	*Boms34*	(CA)_3_CTA(CA)_8_	86(IVR)	58
35	EU489768	*Boms35*	(CA)_48_	-	
36	EU489769	*Boms36*	(CA)_24_	-	
37	EU489770	*Boms37*	(TG)_4_G(TG)_3_	-	
38	EU489771	*Boms38*	(GT)_10_AT(GT)_6_	-	
39	EU489772	*Boms41*	GTAT(GT)_8_GCGTGA(GT)_4_	-	
40	EU489773	*Boms42*	(AT)_3_CC(GT)_3_(AT)_3_(GT)_14_GAGT	-	
41	EU489774	*Boms43*	(CA)_18_C(CA)_3_	-	
42	EU489775	*Boms45*	TAA(CAA)_6_	-	
43	EU489776	*Boms47*	(AG)_12_TG(AG)_8_(TG)_3_(AG)_10_	-	
44	EU489777	*Boms48*	(TC)_5_C_8_G(CT)_4_C_5_G(CT)_5_C_5_G(CT)_6_CCTCG(CT)_8_	-	
45	EU489778	*Boms49*	(CA)_3_CT(CA)_3_CT(CA)_3_CT(CA)_9_	-	
46	EU489779	*Boms50*	(GA)_18_N_4_(GA)_2_G_4_(GA)_2_G_4_(GA)_14_CA(GA)_2_TA(GA)_5_	-	
47	EU489780	*Boms53*	T_8_GT_10_GT_7_CGT_9_GT_6_	-	
48	EU489781	*Boms55*	(AG)_13_GG(AG)_3_GC(AG)_8_	-	
49	EU489782	*Boms58*	A_6_CA_3_GCA_6_TA_5_CA_5_	-	
50	DQ078250	*Boms59*	TGTA(TG)_10_	-	
51	DQ078251	*Boms60*	(CAAA)_2_ A_6_CA_3_GCA_6_TA_5_CA_4_N_26_A_2_GA_9_CGA_4_	-	
52	DQ078252	*Boms61*	T_23_G_2_T_3_GT_3_GT_2_GTAAT_4_C_2_T_5_CTGT_5_	-	
53	EU489783	*Boms62*	A_11_CA_11_CATCACA_4_GA_2_GA_8_	-	
54	EU489784	*Boms63*	A_3_CA_3_CCA_18_	-	
55	EU489785	*Boms64*	CAGA(CA)_2_C(CA)_4_N_12_(CA)_4_C(CA)_2_ (CA)_5_C(CA)_4_C(CA)_4_ACACC(CA)_3_C(CA)_3_	-	
56	EU489786	*Boms68*	T_8_	-	
57	EU489787	*Boms69*	(A/G TT)_4_N_4_T_7_(CTT)_2_AGT_4_CA_2_T_4_GT_4_	-	
58	EU489788	*Boms70*	(GT)-rich	-	

*Boms1–34*: Microsatellite loci isolated from total, small-insert DNA libraries

*Boms35–70*: Microsatellite loci isolated from enriched libraries

T: annealing temperature for *in situ *hybridization

(-): not tested

N: bases that do not present any motif

A significant predominance of interrupted (60.5%) over perfect motifs (34.2%) was observed in both isolation approaches, while only a few (5.2%) were compound. These percentages are quite similar to those observed in *C. capitata *[28] and *B. terrestris *[42]. On the other hand, they are not in agreement with results from *B. tryoni *[43], *B. morii *[44], *D. pseudoobscura *[45] and a recent study in *C. capitata *(Stratikopoulos et al., submitted). Therefore, it is unclear whether these results represent the actual structure of microsatellites in the olive fly genome, since data from closely related species are conflicting. Possibly these results can be attributed to differences in isolation strategies.

### *In situ *hybridization to polytene chromosomes

Cytological analysis of *B. oleae *has revealed five chromosomes (10 polytene arms) and a heterochromatic mass, corresponding to the five autosomes and the sex chromosomes, respectively (for a review see [21]). Well-defined polytene maps have been produced, providing the opportunity for a cytologic localization of molecular markers on the chromosomes.

**Figure 1:**
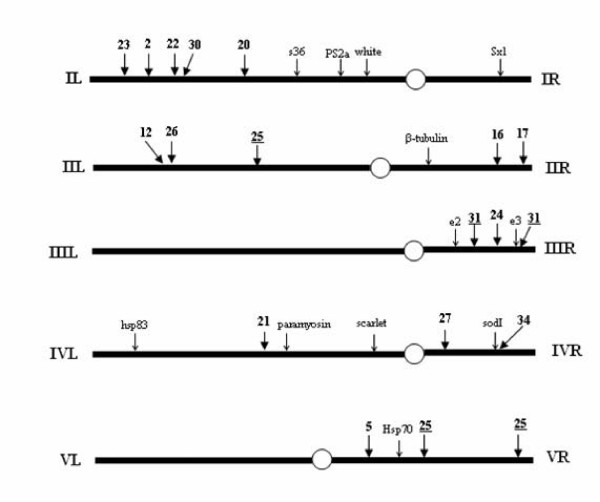
**Schematic representation of the *in situ *localization of microsatellite markers on the polytene chromosomes of *Bactrocera oleae***. Arrows that originate from numbers in bold stand for the Boms microsatellite markers. Underlined numbers refer to microsatellite markers that give multiple signals. All other arrows refer to previously mapped loci [Zambetaki et al 1999].

Twenty of the isolated microsatellite clones were *in situ *hybridised to the salivary gland polytene chromosomes of *B. oleae*, in order to identify their chromosomal localization. At hybridisation temperature of 58°C, sixteen of the microsatellite probes gave specific signals (Table 1) and 13 of them mapped to unique chromosome loci. Clone *Boms20 *hybridised to two neighbouring bands of the same chromosome region, *Boms31 *hybridised to two regions on the same chromosome arm, while *Boms25 *mapped to three regions on two chromosome arms (Table 1, Fig. 1) [Note that microsatellite loci and clones' names are written in italics whereas microsatellite markers' names are written in regular font]. These microsatellite clones gave the same multiple hybridisation pattern even at the higher hybridisation temperature of 62°C. Chromosome localization was not possible for four of the microsatellite probes, although tested at several hybridisation temperatures. Lack of hybridization signal can be attributed either to insufficient hybridization due to small probe length or to the fact that these clones may lie in heterochromatic regions (such as sex chromosomes or centromeric regions). *Boms33 *gave no detectable signal, while the remaining three gave multiple signals.

**Figure 2:**
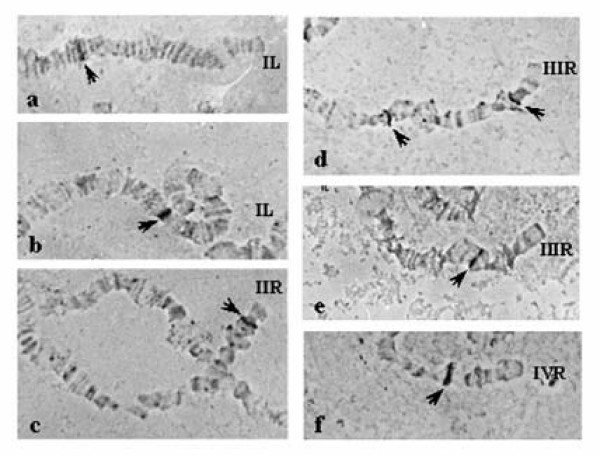
***In situ *hybridization of several microsatellite probes on the salivary gland polytene chromosomes of *Bactrocera oleae***. a: Boms23; b: Boms2; c: Boms17; d: Boms31; e: Boms24; f: Boms34. Arrows indicate the hybridization signals.

The thirteen microsatellites that uniquely mapped to the polytene chromosomes of *B. oleae *are dispersed on seven polytene arms, establishing genetic markers for all five autosomes. Table 1 summarizes the microsatellite hybridization sites and Figure 1 schematically presents the relative positions of the hybridization signals to the polytene chromosome arms of *B. oleae *together with previously described markers [21]. Hybridization signals are presented in Figure 2.

Although their number is small, they enrich the already existing cytological map and are the basis for a low-resolution cytogenetic map that will facilitate future genome projects of the species. It is encouraging that these thirteen markers are dispersed in seven of the ten chromosome arms (except IR, IIIL, VL). However, our *in situ *hybridization data are still limited to support a claim of a uniform distribution of microsatellite loci in the olive fly genome.

### Development of microsatellite markers

Unique sequences flanking each repeat array were used to design PCR primer pairs for the amplification of 42 microsatellites. Thirty-one primer pairs amplified a product of the expected size, as revealed by agarose gel electrophoresis (Table 2). Subsequently, all primer pairs that amplified a specific band were used for the genotyping of 20 individual wild flies (from Greece and Cyprus) and/or up to 37 individuals of a laboratory strain. In addition, 19 *C. capitata *microsatellite markers (Stratikopoulos et al., submitted) that cross-amplified in the olive fly were used, raising the total number of functional primer pairs to 50 (Table 2). In total, 37 primer pairs (29 designed for the olive fly and eight for the medfly) amplified a polymorphic and easily scorable PCR product, while eight pairs amplified a monomorphic one. The five remaining primer pairs generated PCR products that were not easily scored (shuttered or multiple bands or faint signal).

**Table 2:** Microsatellite markers' characteristics and genetic variability

**Marker**	**Primer pairs**	**E.S**	**Tm**	**Samples**	**N**	**n_a_**	**n_e_**	**Ho**	**He**	**HWEG**^2^
Boms2	F: GCTGTTTTGAATGTCAGCATC R: TGTATGCGTGACTGTTTACG	128	50	wild laboratory	20 13	3 3	1.75 2.15	0.45 0.54	0.44 0.56	- -
Boms3a	F: CAGTCGCCCTTTAATTTGC R: GGGTCCTTTTGTTCTCAGG	176	50	laboratory	32	4	2.39	0.63	0.59	-
Boms3b	F: AGGACCCTGGCACAATTCG R: TATGGCATGGCAAGCAGC	171	50	laboratory	32	4	2.26	0.59	0.57	-
Boms5	F: TCTCGCCCCAATTACCAC R: GAATTTTGGCAACATCCAAGC	105	50	laboratory	34	4	3.25	0.79	0.70	-
Boms6a	F: TCACTAAAAGGAGTCCGCAC R: GAGCAGGTCAGAGGCAAAAG	173	50	laboratory	35	3	1.64	0.43	0.39	-
Boms6b	F: AAACCTTACCCTTTTGCCTC R: AGTGCCAACTGAATGCTG	118	50	laboratory	35	3	2.10	0.49	0.53	-
Boms8	F: TGACATACATGCCTTCATTCAC R: CAGAAAAGCTTAAAACTAGCGG	75	50	wild laboratory	20 9	6 3	3.10 2.66	0.85 0.56	0.69 0.66	- -
Boms10	F: CAGAGCATCTCGCTTTGG R: TCAACAATCCCAGCAAAATC	172	50	wild laboratory	20 33	5 2	3.33 1.27	0.70 0.12	0.72 0.22	- +
Boms11	F: ATAGGCATTGGCAGCGAAG R: CACAGTGGGCCGAAATCAC	185	50	wild laboratory	20 25	4 2	1.88 1.68	0.50 0.32	0.48 0.41	- -
Boms12	F: CGCGTTTTCATACTTTAACACC R: TTCATTTGGCCTTTGTGC	158	50	wild	19	3	1.17	0.16	0.15	-
Boms14	F: TTTGTAATTCGCAGAAGGCAC R: AGGAGGACTGACAGAAGGACAC	147	50	wild	8	4	2.72	0.38	0.68	
Boms16	F: CAGACAATGGATGGATACATGC R: GGAGAAGTCAAATTGTGACAGC	109	50	wild	20	5	1.72	0.50	0.43	-
Boms17	F: ATTAGACCATAGTGTTCTCAC R: AAGATGTTGAGTGCCGTTG	170	50	wild laboratory	20 31	7 5	5.76 1.41	0.35 0.19	0.85 0.29	+ -
Boms18	F: GCCATGAATGCAGACCAC R: CCTATTCAAATGCACGCAAAAC	171	50	wild laboratory	20 33	6 3	2.56 1.94	0.80 0.39	0.63 0.49	- +
Boms21	F: TCGCCTCTTACCTCACAACC R: ACCATCCTTAGTCAGCACAGTC	188	50	wild laboratory	20 28	6 4	3.77 1.16	0.75 0.14	0.75 0.14	- -
Boms22	F: GTAAAGCACACGGAAGCG R: TGAGGTCAAAAAGGATGCTAAG	211	50	wild laboratory	18 7	2 2	1.06 1.32	0.06 0.29	0.06 0.26	- -
Boms24	F: ATTTCGCTTGCCACAAAC R: CGCCCAAGCACTTAAAAC	215	50	laboratory	33	2	1.77	0.39	0.44	-
Boms25	F: TGGAATGCGCTATTTTGTTG R: ACTCGTATATACGTACATGG	167	50	wild laboratory	20 33	5 3	3.49 2.16	0.80 0.55	0.73 0.55	- -
Boms27	F: CGACTTGAAGGACAATTGG R: GGCGTGAGTAGTTTCTATAAGC	129	50	wild laboratory	20 10	5 3	2.02 2.41	0.55 0.50	0.52 0.62	- -
Boms29	TGAAGGTGATGAATGAAAGC GGAATGACTGTGAGCAAGC	118	50	wild laboratory	20 13	5 1	2.57 1.00	0.60 0.00	0.63 0.00	-
Boms30	F: CTGACTTCTTGCTTTACACG R: CAGCTTATCTGCTTTAAGTGC	150	50	wild laboratory	20 9	4 4	2.12 2.19	0.70 0.78	0.54 0.58	-
Boms31	F: TGCTTGAGTTGCTCGTTGG R: GCCGCATGACATAAAGAATCG	173	50	wild laboratory	20 30	4 2	3.27 1.03	0.75 0.03	0.71 0.03	-
Boms32	F: TGTATGTATTTGTGCGTCG R: GCTTAGACCATTTGCTCC	125	50	wild	20	7	3.96	0.55	0.77	-
Boms34	F: ACGCCGCACACTTCTTAAAC R: CACCCAACTTTTGTAGTTTCC	219	50	laboratory	34	3	2.08	0.65	0.53	-
Boms47	F: CAAACACACGCTAAAACG R: TTTAACCCAGAGGCTTGC	158	50	wild	18	6	3.15	0.61	0.70	-
Boms58	F: AGTTGGACGCGCACATATC R: AGCGCGTACGAGCTTTAGC	181	50	wild laboratory	18 30	7 3	5.02 1.15	0.72 0.13	0.82 0.13	- -
Boms59	F: AGCGCTTACATAAATATAGCTAC R: TCCCCGTAAAGCCATAAAGTC	171	50	wild	20	5	2.27	0.50	0.57	-
Boms60	F: TGGACGCGCACATATCAG R: ACGACGTTTAGCGGAAATGAG	170	50	wild laboratory	20 37	6 3	3.14 1.21	0.70 0.19	0.70 0.18	- -
Boms62	F: CTTTCGCTGCCTCCATTTG R: CAAAACCCCTCTGCAATCC	174	50	wild	20	2	2.00	0.55	0.51	-
Boms64a	F: TGCTAGGCTGAACATTCG R: TGTTTTGCTGTTTCCAGG	129	50	wild	20	Multiple bands				
Boms64b	F: TGGAAACAGCAAAACACC R: AGCGAATCAAGAGACAGC	137	50	wild	10	Multiple bands				
Medflymic9	Stratikopoulos et al., submitted		50	wild	8	3	2.42	0.88	0.63	
Medflymic142	>>		50	wild	9	monomorphic				
Medflymic149	>>		50	wild	8	monomorphic				
Medflymic150	>>		50	wild	8	3	2.03	0.50	0.54	
Medflymic151	>>		50	wild	20	5	2.66	0.35	0.64	+
Medflymic152	>>		50	wild	20	6	5.00	0.40	0.82	+
Medflymic153	>>		50	wild	20	3	2.24	0.40	0.57	-
Medflymic154	>>		50	wild	10	monomorphic				
Medflymic157	>>		50	wild	10	monomorphic				
Medflymic158	>>		50	wild	8	3	2.25	0.63	0.59	
Medflymic163	>>		50	wild	10	monomorphic				
Medflymic22	>>		55	wild	20	Multiple bands				
Medflymic23	>>		55	wild	20	monomorphic				
Medflymic29	>>		55	wild	20	Multiple bands				
Medflymic40	>>		55	wild	20	Multiple bands				
Medflymic61	>>		55	wild	20	4	2.32	0.45	0.58	-
Medflymic64	>>		55	wild	20	monomorphic				
Medflymic72	>>		55	wild	20	monomorphic				
Medflymic109	>>		55	wild	20	2	1.05	0.05	0.05	-

	Mean wild (monomorphic excluded) Mean laboratory (monomorphic excluded)				19.5 26.5	4.63 3.14	2.74 1.90	0.53 0.44	0.57 0.44	

E.S: Expected size

Tm: PCR annealing temperature

N: sample size

n_a_: actual number of alleles; n_e_: effective number of alleles

Ho: heterozygosity observed; He: heterozygosity expected

(-): in HWE; (+): out of HWE

The mean allele number per locus was 4.63 for natural populations and 3.14 for laboratory strains (monomorphic loci excluded), demonstrating their usefulness in population analyses of the species. Conformation to HWE was tested for 26 loci for natural populations and 19 loci for laboratory strains, according to G^2 ^criterion, at a significance level of 5%. Only five deviations were observed due to homozygosity excess, which can be attributed to small sample size or to the presence of null alleles (Table 2).

### Cross – species amplification in Tephritidae

The 29 primer pairs designed for the olive fly and proved polymorphic were tested in a pooled mix of five flies from each one of 11 Tephritidae species. Twenty-six of them amplified a specific DNA fragment, at least in one of the species examined. Four species belong to *Bactrocera *(*B. correcta, B. cucurbitae, B. dorsalis *and *B. tryoni*), four to *Anastrepha *(*A. fraterculus, A. ludens, A. serpetina *and *A. striata*), two to *Ceratitis *(*C. capitata *and *C. fasciventris*) and one belongs to *Rhagoletis *(*R. cerasi*) (Tables 3 and 4).

**Table 3:** Cross-species amplification of *Bactrocera oleae *microsatellite markers in other Tephritidae species

**Species**	**Bactrocera**					**Anastrepha**				**Ceratitis**		**Rhagoletis**
**Markers**	*Bo*	*Bcu*	*Bco*	*Bd*	*Bt*	*Af*	*Al*	*Astr*	*Aser*	*Cc*	*Cf*	*Rc*
Boms2	150	400	400			Sm	450/ Sm	Sm	Sm	200	200f	Sm
Boms3a	210	210	210	210	230					190	190	500
Boms3b	200	X	220	200	210	Sm	Sm	Sm	X	150		200
Boms5	110	500		700	130				300			
Boms6a	150/ 400	350/ Sm		350	300/ 500			Sm	Sm		600	200/ 300
Boms6b	150										150f/ 200	
Boms8	75										200	
Boms10	200			180	200		180/ Sm		120f/ 150f	150	200/ 300	300
Boms11	200	Sm	220	Sm	Sm	Sm	Sm	Sm	Sm	Sm	Sm	500
Boms12	200							Sm	Sm			
Boms14	150		150		150	300		Sm	Sm			
Boms16	100			100	100							
Boms18	171		170	170	190	Mb	Mb	Mb	Mb	300	300	
Boms21	188	190	180		180							
Boms22	210				500f							
Boms24	200		250	250	250							
Boms27	130		130	130	130	Sm	Sm	Sm	Sm	Sm	150/ Mb	Sm
Boms29	120											
Boms30	150	150	150	150	150		Mb	130	130	110	110	110
Boms31	170		120	170	170	500	120f/ 300/ 500/ 600	120f	120f/ 400/ 500/ 700		800	450f
Boms32	150	150										
Boms34	200		200	X	200							
Boms58	180		400	150f/ 180f	150f/ 180f	400	400/ 500	500	400	Sm	Sm	Sm
Boms60	170	150f	170	170	170	150	150	150	150	170	250/ Mb	150
Boms62	174	200	160	200	180	160/ Mb	160/ Mb	160/ Mb	160/ Mb	200	160	
Boms64a	150							300/ 450	500			

Numbers in columns indicate PCR product size, as revealed by agarose gel electrophoresis

Sm: smear; f: faint band; Mb: multiple bands

Bo: B. oleae; Bco: B. correcta; Bcu: B. cucurbitae; Bd: B. dorsalis; Bt: B. tryoni; Cc: C. capitata; Cf: C. fasciventris; Af: A. fraterculus; Al: A. ludens; Aser: A. serpentina; Astr: A. striata; Rc: R. cerasi

**Table 4:** Conservation of *Bactrocera oleae *microsatellite markers in Tephritidae

Species/ **Genera**	Functional primer pairs	Expected size	Presence of SSR motif	Presence of the expected motif	Mean number of uninterrupted repeats
*B. oleae*	29	29	29	29	100%
*B. correcta*	15/29 (51.7%)	13/15 (86.7%) 13/29 (44.8%)	6/7 (85.7%)	5/6 (83.3%) 5/7 (71.5%)	20/50 (40%)
*B. cucurbitae*	9/29 (31%)	6/9 (66.7%) 6/29 (20.7%)	2/2 (100%)	2/2 (100%) 2/2 (100%)	11/27 (40%)
*B. dorsalis*	14/29 (48.3%)	12/14 (85.7%) 12/29 (41.4%)	4/4 (100%)	4/4 (100%) 4/4 (100%)	29/36 (80.5%)
*B. tryoni*	19/29 (65.5%)	17/19 (89.5%) 17/29 (58.6%)	5/6 (83.3%)	5/5 (100%) 5/6 (83.3%)	32/53 (60%)

**Bactrocera** **genus**	**57/116 (49.1%)**	**48/57 (84.2%)** **48/116 (41.4%)**	**17/19 (89.5%)**	**16/17 (94.1%)** **16/19 (84.2%)**	**92/166 (55.4%)**

*A. fraterculus*	5/29 (17.2%)	2/5 (40%) 2/29 (6.9%)	2/2 (100%)	1/2 (50%) 1/2 (50%)	8/9 (88.9%)
*A. ludens*	7/29 (24.1%)	5/7 (71.5%) 5/29 (17.2%)	1/1 (100%)	1/1 (100%) 1/1 (100%)	11/9 (122%)
*A. serpentina*	9/29 (31%)	6/9 (66.7%) 6/29(20.7%)	1/1 (100%)	1/1 (100%) 1/1 (100%)	8/9 (88.9%)
*A. striata*	7/29 (24.1%)	5/7 (71.5%) 5/29 (17.2%)	1/1 (100%)	1/1 (100%) 1/1 (100%)	12/9 (133%)

**Anastrepha genus**	**28/116 (24.1%)**	**18/28 (64.3%)** **18/116 (15.5%)**	**5/5 (100%)**	**4/5 (80%)** **4/5 (80%)**	**39/36 (108.3%)**

*C. capitata*	8/29 (27.6%)	7/8 (87.5%) 7/29 (24.1%)	1/2 (50%)	1/1 (100%) 1/2 (50%)	2/3 (66.7%)
*C. fasciventris*	12/29 (41.4%)	9/12 (75%) 9/29 (31%)	2/2 (100%)	2/2 (100%) 2/2 (100%)	12/24 (50%)

**Ceratitis genus**	**20/58 (34.5%)**	**16/20 (80%)** **16/58 (27.6%)**	**3/4 (75%)**	**3/3 (100%)** **3/4 (75%)**	**15/27 (55.5%)**

***R. cerasi***	**8/29 (27.6%)**	**4/8 (50%)** **13.8%**	**1/3 (33.3%)**	**1/1 (100%)** **1/3 (33.3%)**	**2/3 (66.7%)**

*Functional primer pairs*: number of primer pairs that cross-amplified successfully; *Expected size*: number of primer pairs that cross-amplified and produced fragment of the expected size (calculated either in regard to total available primer pairs or in regard to primer pairs that successfully cross-amplified); *Presence of SSR motif*: number of cross-species amplification products that harboured a microsatellite motif; *Presence of the expected motif*: number of cross-species amplification products that harboured the expected microsatellite motif (calculated either in regard to total sequencing reactions or in regard to cross-species amplification products that harboured a microsatellite motif); *Mean number of uninterrupted repeats*: sum and comparison of the number of uninterrupted repeats for primer pairs that cross-amplified and harboured the expected motif. Dividend represents repeats in the new species, while divider stands for number of repeats in *B. oleae*

**Figure 3:**
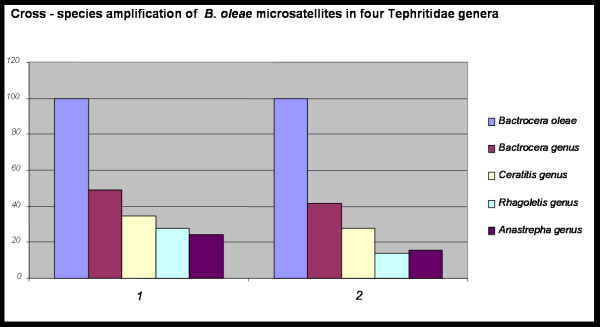
**Cross-species amplification of *Bactrocera oleae *microsatellites in four Tephritidae genera**. 1: Percentage of cross-amplified primer pairs. 2: Percentage of primer pairs that produced a fragment of the expected size.

A total of 113 PCR products were amplified. The species with the highest degree of amplification was *B. tryoni *(19/29), while with the lowest was *Rhagoletis cerasi *(8/29). As expected, the highest percentage of amplification was inside *Bactrocera*, with a mean of 49.1%. *Ceratitis *presented the next higher amplification degree (34.5%), followed by *Rhagoletis *and *Anastrepha *(27.6% and 24.1%, respectively) (Table 4 and Figure 3-1). It is worth mentioning that *B. cucurbitae *exhibited very low amplification rate, similar to that of *Anastrepha*. Finally, *C. capitata *presented substantially lower degree of amplification than *C. fasciventris*.

The majority of PCR products had similar size (less than ~50 bp difference, as estimated by agarose gel electrophoresis) with those obtained in *B. oleae *(about 76%). Still, the highest degree of PCR product size conservation was inside *Bactrocera *(84.2%), although *Ceratitis *showed a comparable percentage (80%). *Anastrepha *and *Rhagoletis *presented significantly lower values (64.3% and 50%, respectively) (Table 4 and Figure 3-2). Surprisingly, *B. cucurbitae *showed very low size conservation (66.7%), comparable to that of *Anastrepha*, implying that the low amplification value mentioned before may not be a PCR artefact. This in not the case in *C. capitata*, since size conservation is very high (87.5%). This value is higher than that of *C. fasciventris *and comparable to that of *Bactrocera*, suggesting that medfly's low amplification value is more likely a PCR artefact.

### Analysis of cross-species amplification products

Amplification of a band of expected size does not necessarily mean that the expected microsatellite motif is also present. To evaluate the degree of motif conservation, 31 of the reactions that produced a specific band were subcloned and sequenced. We focused on PCR products of similar to the expected size and distributed in as many species as possible. Twenty-seven of the amplification products harboured a repeat motif, 25 of which contained the same as that of *B. oleae*. Six of the products harboured new motifs (instead of or in addition to the expected ones) (Table 5).

**Table 5:** Microsatellite loci obtained through cross-species amplification

Locus	*B. oleae *motif	Motif of other Tephritidae
*Boms3a*	(GT)_14_	*Bt*: (AT)G_2_(AT)_3_AC(AT)A_2_(AT)**(GT)**_2_(AT)/**(TG)**_2_(TA)_2_**(TG)**_8_ *Bco*: **(TG)**_2_TA(GT)N_7_**(TG)**_2_(TA)/**(TG)**(GA)**(TG)**_2_(TA)_2_**(TG)**(TA)_3_(TA_3_)_2_**(TG)**_3_C**(GT)**_3_ *Bcu*: (TA)_3_A(TA)CA_2_**(GT)**_4_ *Bd*: (TA)_2_**TG**(TA)CA(TA)_3_A_2_**(TG)**_5_CA**(TG)**(TC)_2_(TA)_2_**(TG)**_7_ *Cf*:**(TG)**_4_T_2_**(TG)**_8_
*Boms3b*	(CAA)_3_CAG(CAA)_3_/ (CAA)_2_CAG(CAA)	*Bco*: **(CAA)**_2_AAA**(CAA)/(CAA)**_3_ *Bd*: **(CAA)**_2_AA**(CAA)/(CAA)**_5_ *Bt***: (CAA)**_2_A**(CAA)**/**(CAA)**_3_ *Cc*:/**(CAA)**A_3_**(CAA)**_2_ *Rc*: A_4_CA_2_CGATACA_5_N_9_*A*_5_/**(CAA)**G**(CAA)**_2_(CAG)
*Boms10*	(CA)_10_GA(CA)_2_	*Cc*: - *Cf*: **(CA)**GAC**(CA)**_4_ *Rc*: -
*Boms11*	(CA)_7_CG(CA)_3_	*Bco*: - *Rc*: -
*Boms14*	(AT)_2_GT(AT)_11_	*Bco*:**(TA)**_2_G**(TA)**CA**(TA)**_3_A_2_ *Af*: *G*_6_N_7_(GT)_3_
*Boms16*	(CA)_10_	*Bco*: *T*_5_ATCA_4_/*A*_5_TCA_2_A_2_ *Bd*: **(CA)**TG**(CA)**_4_ *Bt*: **(CA)**TA**(CA)**_4_CG**(CA)**
*Boms21*	(GT)GG(GT)_13_AT(GT)	*Bco*: (GA)_3_**(GT)**_6_ *Bcu*: (AT)_3_**(GT)**(AT)**(GT)**_7_ *Bt*: (GA)_3_**(GT)**_3_AT**(GT)**_5_AT**(GT)**
*Boms60*	CACA_2_(CA_3_)_2_ A_6_CA_3_GCA_6_TA_5_CA_4_N_26_A_2_G**A**_9_CGA_4_	*Bco*: CACA_2_(CA_3_)_2_/A_5_C_2_A_3_(GT)_2_A_4_G_2_**A**_5_N_7_A_5_N_5_A_3_CA_3_CA_2_CA_3_N_4_A_3_N_3_A_2_N_3_**A**_5_GA_2_CGA_4_ *Bd*: CACA_2_(CA_3_)_2_/**A**_13_CA_6_TA_4_N_4_A_3_CA_2_TA_2_CA_5_ *Bt*: CACA_2_(CA_3_)_2_/A_6_CA_3_GCA_6_TA_5_CA_5_N_28_A_2_G**A**_9_CGA_4_ *Af*: CACA_2_(CA_3_)_3_/*(GA)*_4_A_3_G**A**_8_GA_4_N_9_GA_3_GATA_4_T**A**_8_GACA_5_CA_4_ *Al*: CACA_2_(CA_3_)_3_/*(GA)*_4_A_3_GA_9_GA_4_N_9_CA_3_GATA_4_T**A**_11_CA_5_CA_5_ *Aser*: CACA_2_(CA_3_)_3_/CA_5_GA_5_GA_4_TACA_4_TACA_2_TCA_2_CA_3 _GATA_4_TA_4_TACA_5_C**A**_8_ *Astr*: CACA_2_(CA_3_)_3_/*(GA)*_4_A_3_GA_6_C_2_A_3 _TACA_2_TCA_2_CA_3 _GATA_4_TA_9_C**A**_12_
*Boms64a*	CAGA(CA)_2_C(CA)_4_N_12_(CA)_4_C(CA)_2_	*Bt*: **(CA)**_2_A**(CA)**_2_C(CA)T_2_**(CA)**_2_

In bold: cases of preservation of the expected motif

N: non-motif bases

Bco: B. correcta; Bcu: B. cucurbitae; Bd: B. dorsalis; Bt: B. tryoni; Cc: C. capitata; Cf: C. fasciventris; Af: A. fraterculus; Al: A. ludens; Aser: A. serpentina; Astr: A. striata; Rc: R. cerasi

Nineteen (of the 31) sequencing reactions were performed in *Bactrocera*. The presence of a microsatellite motif in 18 of them (16 of which had the expected motif), demonstrates their potential in the analysis of other *Bactrocera *species. Results from other genera are encouraging, although preliminary. In *Ceratitis*, for example, four sequencing reactions were performed, three of which exhibited the expected motif. In *Anastrepha*, five sequencing reactions were performed, all of which exhibited a microsatellite motif with four cases possessing the expected one (however, they all refer to the same locus in four different species). Finally, in *Rhagoletis*, three sequencing reactions were performed, one of which exhibited a microsatellite repeat of the expected motif. These results are summarized in Tables 4 and 5 and demonstrate the potential utility of these markers in the analysis of Tephritidae genera other than *Bactrocera*.

Mean number of uninterrupted repeats was measured only in cases where the expected motif was present in cross-species amplification products (Table 4). In seventeen cases within *Bactrocera *(regarding seven microsatellite loci), the mean number of uninterrupted repeats was 9.8 for *B. oleae *and 5.4 for the other *Bactrocera *species. Same analysis for three PCR products (regarding three microsatellites) in *Ceratitis *gave a mean of 9.0 and 5.0 uninterrupted repeats for *B. oleae *and *Ceratitis*, respectively. Although sequencing data are still limited, it is obvious that microsatellites tend to present longer arrays in the species in which they were isolated from. This has been described in a variety of species, such as *Drosophila *[40,46] and primates [47], and has been attributed to the fact that microsatellites can evolve directionally and at different rates in closely related species.

### Sequencing analysis and phylogenetic comparisons

Although we did not perform a phylogenetic analysis, it seems that measures of cross-species amplification (e.g., percentages of functional primers and expected size of PCR products) are indicative of the phylogenetic history of these species. Our results support the notion that three of the Bactrocera species are very close to *B. oleae*, while the fourth (*B. cucurbitae*) seems to be more distant (Table 4, Figure 3). Also, *Ceratitis *seems to be more closely related to *Bactrocera *than *Anastrepha *and *Rhagoletis *seems to be the most distantly related genus to *Bactrocera*. These results perfectly replicate the exact same relationships observed in the most recent phylogenetic analysis of these species based on mtDNA sequencing data [48]. Secondarily, they are also supported by several other studies from different insect species based on alignment of mitochondrial 16S rDNA sequences [49,50] and 18S rDNA sequences [51], which show that *Bactrocera *is more closely related to *Ceratitis*, and closer to *Anastrepha *than it is to *Rhagoletis*. In addition, we also performed sequencing alignments of a few cross-species amplification products of some of our markers (data not shown). In all cases, the different species were clustered to their respective genera with high bootstrap values. Although these data are very limited, they come from dispersed regions of nuclear DNA which gives significant value to phylogenetic analyses. There are studies supporting that microsatellite data can shed light to phylogenetic relationships among closely related taxa [52-54]. Sequencing analysis of more microsatellite markers can probably reveal complex phylogenetic relationships among different Tephritidae species, especially in cases of species complexes.

### Polymorphism of cross-species microsatellite markers

Presence of a microsatellite motif does not necessarily mean that these loci can be used as genetic markers. Nineteen microsatellite markers developed in the medfly cross-amplified in the olive fly (Table 2). The fact that eight of them were polymorphic in a relative small sample (twenty wild flies) confirms the possible utility of the markers presented here in the analysis of other Tephritidae species.

## Conclusion

Since their discovery, microsatellite markers have been particularly useful in population and genetic analyses, mainly due to their high degree of polymorphism. Their significance is even greater in organisms like the olive fly, where the lack of morphological markers makes classical genetic analysis practically impossible. The interest in olive fly's genetics is not only theoretical, since modern genetic and molecular tools have benefited several operational SIT programmes, particularly those where GSSs are involved [15]. The observed polymorphism of the developed microsatellite markers (both in laboratory and natural populations) guarantees their utility in genetic and population analyses. A subset of these markers has already been successfully used in previous population studies [36,19]. The existence of well-described polytene chromosomes in the olive fly [21] and the possibility of cytological localization of molecular markers by *in situ *hybridisation provide a powerful method to link the genetic and molecular information of an organism. The existence of defined polytene chromosomes in other Diptera [55,56] also offers the opportunity to establish syntenic linkages and to study the evolutionary relationships of separate chromosomal segments [57,21]. Cross-species amplification of the developed markers to other Tephritidae demonstrates their potential utility in those species. Sequencing analysis of several cross-amplified products revealed a varying degree of conservation that declines outside the *Bactrocera *genus. Such sequencing analyses can also assist the clarification of phylogenetic relationships among different species, particularly in cases of species complexes.

## Methods

### Fly culture and stocks

Field-collected samples: Olive fruits were collected and kept in the laboratory until adult flies emerged. These flies were preserved individually at -20°C until DNA extraction.

#### Laboratory strain

*B. oleae *flies used for *in situ *hybridisation and polymorphism analysis were obtained from the Department of Biology, "Demokritos" Nuclear Research Center, Athens, Greece. In our laboratory the stock was reared on an artificial medium based on yeast hydrolysate, sucrose, egg yolk and water [58-60] at 25 ± 1°C and a 12 h light: 12 h dark cycle.

### Construction and screening of total small insert genomic libraries

Genomic DNA was extracted from adult flies of the laboratory strain as described in [61]. Approximately 3 μg of genomic DNA were digested to completion with *Mbo*I and digestion products were electrophoresed in 1% agarose gel (Seakem GTG). Restriction fragments that ranged between 500 bp and 1200 bp were isolated from the gel (Jetquick gel extraction kit, Genomed) and cloned into the *BamHI *site of plasmid vector pBlueskript II SK (Stratagene). About 10^4 ^recombinant clones were transferred onto nylon membranes (Hybond-N, Amersham), screened with a mix of radioactively labeled (CT)_15 _and (GT)_15 _oligonucleotides. Labelling was performed with terminal transferase (Promega), under the conditions suggested by the manufacturer. Hybridisation was performed at 48°C in standard hybridisation solution (6× SSC, 0.5% SDS, 5× Denhardt's) for at least 16 hours. Membranes were then washed twice for 5 min in 2× SSC/0.1% SDS at 25°C and once for 15 min in 1× SSC/0.1% SDS at 37°C and subsequently exposed with film. Positive colonies underwent a secondary screening and plasmid DNA was then purified by the alkaline lysis method [62] and electrophoresed. Clones of convenient size inserts (i.e., 500–1000 bp) were sequenced (Thermo Sequenase core Sequencing kit, Amersham). Sequencing reactions were analysed in an automatic sequenator and the microsatellite repeat motif was determined.

### Construction of microsatellite-enriched genomic libraries

Genomic DNA was extracted as above. Enriched libraries were prepared according to [63]. Seven libraries were constructed using different oligonucleotide probes [(GA)_15_, (CA)_15_, (GT)_15_, (CT)_15_, (AT)_15_, (GC)_15 _and (GAC)_10_]. Two rounds of enrichment were performed for each library. Enriched products were cloned either in plasmid vector pBlueskript SKII digested with *EcoRI*, (without removal of the amplification linkers), or into the *BamHI *site of the pUC18 vector (Ready-To-Go™ pUC18/*BamHI*, Amersham), (after linker removal). Insert size of recombinant clones was estimated on agarose gels and selected clones were sequenced as above. Selection was done either at random, or after Southern transfer and hybridization with (GT)_15 _and (CT)_15 _radiolabelled probes.

### *In situ *hybridization procedures

Squash preparations of salivary gland chromosomes were made from 10–12 day-old third instar larvae and 1–2 day old pupae, as previously described [64]. Microsatellite containing clones were labelled with digoxigenated dUTP (Dig-11dUTP) using the random priming method and *in situ *hybridized to polytene chromosomes according to [64]. Hybridization temperature was 55–62°C (Table 1). Signals were detected with specific antibodies (ROCHE Diagnostics, Mannheim, Germany). Five or more chromosomal preparations were hybridized with each probe and at least ten well-spread polytene nuclei per preparation were examined to identify the hybridization signals.

### Genotyping

PCR amplification was performed in a 10 μl volume that contained ~10 ng of DNA, 1.6 mM MgCl_2_, 1× reaction buffer [Promega: 10 mM Tris-HCl (pH 9.0), 50 mM KCl, 0.1% Triton X-100], 0.2 U Taq polymerase (Promega), 0.2 mM of each dNTP, 3 pmol of each primer. PCR products were subsequently separated in 1.5% agarose gels. For genotyping, PCRs were performed as above with the only difference that one fifth of one of the primers of each pair was end-labeled with [γ^32^P]-ATP, using T4 polynucleotide kinase (MBI, Fermentas) [65]. Amplification was performed on a PTC-100 thermocycler (MJ Research Inc) for 30 cycles of 1 min at 95°C, 1 min at 50°C and 1 min at 72°C. PCR products were electrophoresed on 5% denaturing polyacrylamide gels and visualized by autoradiography.

### Data analysis

Genetic variability was measured as the mean number of alleles per locus, effective number of alleles and observed and expected heterozygosity. Conformation to HWE was tested at a significance level of 5%, according to G^2 ^criterion. All computations were performed with POPGENE version 1.31 software [66].

#### Sequencing of cross-species amplification products

PCR products were electrophoresed, isolated from gel with the 'PCR Clean up and Gel extraction' kit (Nucleospin) and subsequently ligated to the pCR2.1-TOPO vector with the TOPO TA cloning kit (Invitrogen). Recombinant vectors were used to transform *E. coli *competent cells of the XL-1 strain. Plasmid DNA was extracted with the alkaline lysis method, as above and sequence analysis was performed by Macrogen Inc (Korea).

## Authors' contributions

AAA isolated the microsatellite markers, performed most of the analysis described in the manuscript and drafted the largest part of the manuscript. EES participated in the isolation of the microsatellite markers and developed the *C. capitata *markers. ED carried out the *in situ *hybridisations and helped to draft the manuscript. EGK participated in the cross-species analysis. PMT analysed the *in situ *hybridisation results and helped to draft the manuscript. AZ participated in the design of the study, hosted most of the research performed in her laboratory and helped to draft the manuscript. KDM participated in its design and coordination and helped to draft the manuscript. All authors read and approved the final manuscript.
